# [2,2′-Bis(di­phenyl­phosphan­yl)-1,1′-binaphthyl-κ^2^ *P*,*P*′]di­chlorido­platinum(II) acetonitrile trisolvate

**DOI:** 10.1107/S2414314620010482

**Published:** 2020-08-04

**Authors:** Jason D. Braun, Guneet Uppal, David E. Herbert

**Affiliations:** a Department of Chemistry, University of Manitoba, Winnipeg, Manitoba, R3T 2N2, Canada; University of Zürich, Switzerland

**Keywords:** crystal structure, racemate, platinum, metallic complex

## Abstract

The crystal structure (150 K) of the racemic title compound, [PtCl_2_(C_44_H_32_P_2_)]·3CH_3_CN, has been determined. The asymmetric unit comprises a single mol­ecule of the title compound co-crystallized with three aceto­nitrile solvent mol­ecules. Four mol­ecules are observed in the unit cell, with *R* and *S* enanti­omers present in a 2:2 ratio. Evidence of intra­molecular π-stacking is observed with no discernable inter­molecular inter­actions.

## Abstract 2

The crystal structure of racemic di­chloro­[2,2′-bis­(di­phenyl­phosphino)-1,1′-binaphth­yl]platinum(II) has been determined at 150 K. The asymmetric unit consists of a single mol­ecule of the title compound co-crystallized with three aceto­nitrile solvent mol­ecules.

## Graphical abstract

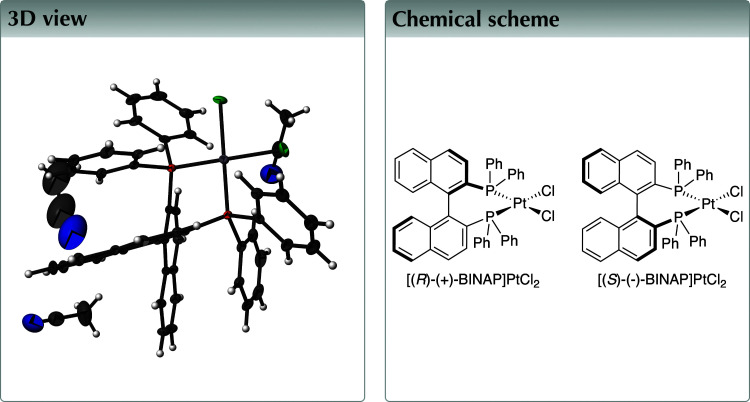

## Structure description

**Figure 1:**
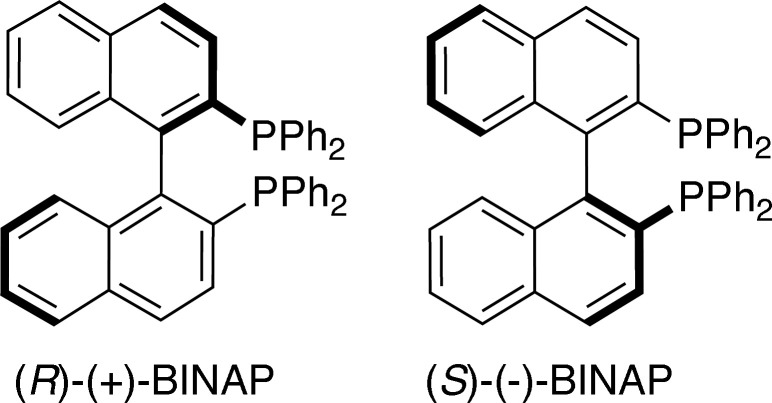
BINAP atropisomers.

**Figure 2:**
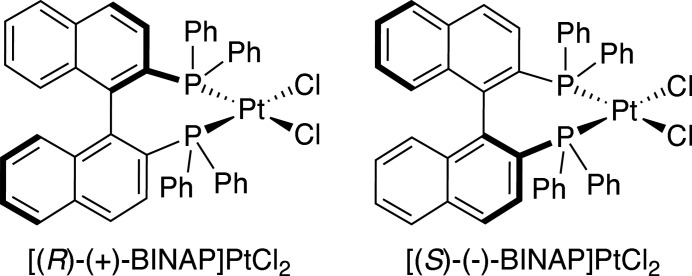
Atropisomers of (BINAP)PtCl2.

The increasing demand for chiral compounds in the pharmaceutical, agrochemical and food industries has driven the development of chiral ligands and coordination complexes, which can perform asymmetric catalysis to yield desirable organic mol­ecules with high enanti­oselectivities (Noyori, 1994 ▸). Popular design motifs for chiral ligands are those that incorporate an atropisomeric backbone featuring *C*_2_ symmetry (Genet *et al.*, 2014 ▸). 2,2′-Bis(di­phenyl­phosphino)-1,1′-binaphthyl (BINAP, Fig. 1 ▸), first developed by Noyori and Takaya in 1980 (Miyashita *et al.*, 1980 ▸), fits this brief. In the decades since its appearance in the literature, BINAP derivatives have been used to construct a wide variety of coordination complexes, typically involving late transition metals (Misra *et al.*, 2017 ▸). Palladium complexes of BINAP have been historically amongst the most common (Pereira *et al.*, 2013 ▸). They are exceptionally popular due to their successful and versatile application as catalysts in a variety of organic reactions such as the enanti­oselective benzoyl­ation of alcohols (Iwata *et al.*, 2002 ▸) and asymmetric alkyl­ations (Guerrero-Ríos & Martin, 2014 ▸). Although less common than complexes of the second-row metal Pd, atropisomers of (BINAP)PtCl_2_ (Fig. 2 ▸) have found use in catalytic reactions such as enanti­oselective Baeyer–Villiger oxidations of cyclic ketones with hydrogen peroxide (Strukul *et al.*, 1997 ▸) and as precatalysts for asymmetric carbonyl-ene reactions (Doherty *et al.*, 2006 ▸). Enanti­omeric complexes of the formula *L*_2_PtCl_2_ including (BINAP)PtCl_2_ have also been examined for their cytotoxic activity against cancer cell lines and their ability to bind to the human telomeric sequence folded in the G-quadruplex structure (Bombard *et al.*, 2010 ▸). There is therefore significant inter­est in elucidating the solid-state structures of these types of compounds to help guide future design strategies appropriate for particular applications.

**Figure 3:**
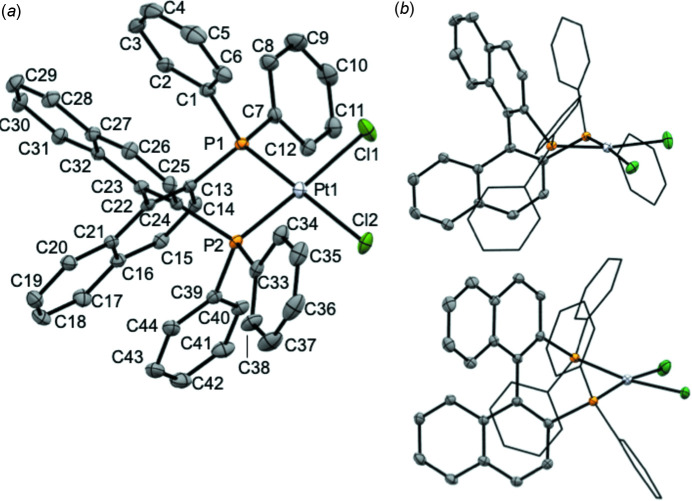
Solid-state structure of (BINAP)PtCl_2_ showing (*a*) fully atom labels of the *R* enanti­omer and (*b*) side-on views of both *R* and S atropisomers present the crystal structure. Displacement ellipsoids are shown at the 50% probability. Hydrogen atoms and co-crystallized aceto­nitrile solvent mol­ecules are omitted for clarity.

**Figure 4:**
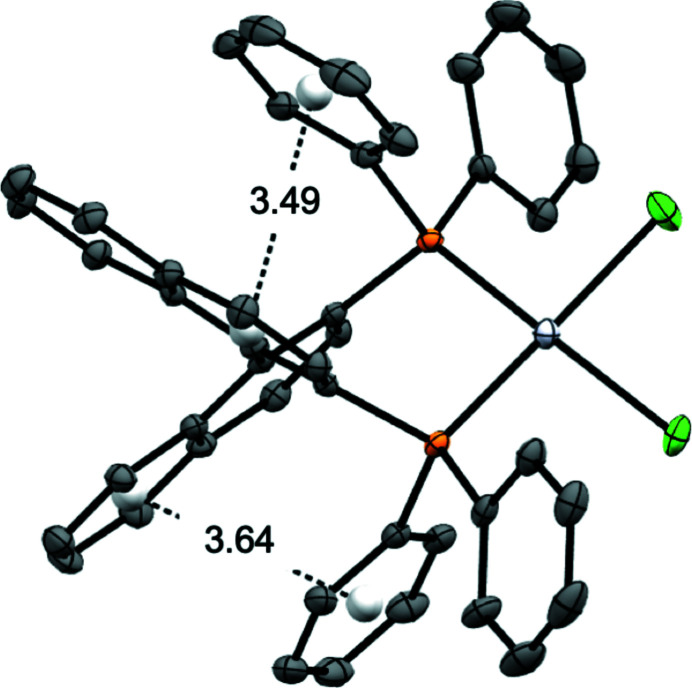
View showing the close intramolecular contacts between the naphthyl and phenyl rings in the title compound.

While the structure of {(*R*)-BINAP}PtCl_2_ has been described as a di­chloro­methane solvate in the ortho­rhom­bic space group *P*2_1_2_1_2_1_ (Doherty *et al.*, 2006 ▸), the corresponding racemate (*rac*BINAP)PtCl_2_ has yet to be structurally characterized. We report here the solid-state crystal structure of (*rac*BINAP)PtCl_2_ determined *via* single-crystal X-ray diffraction and discuss its structural properties. The solid-state structure of [*rac*BINAP]PtCl_2_ obtained by modelling single-crystal X-ray diffraction data is shown in Fig. 3 ▸ with selected bonds and angles in Table 1 ▸. The compound crystallizes in the monoclinic space group *P*2_1_/c with three aceto­nitrile solvent mol­ecules present within the asymmetric unit. The complex adopts a slightly distorted square-planar coordination geometry about the central Pt^II^ atom with *trans* atoms situated at bond angles of 171°, resulting in a τ_4_ value of 0.12. The bidentate BINAP ligand coordinates to Pt with a bite angle (P1—Pt1—P2) of 92.87 (3)°, consistent with typical literature values of approximately 93° (Birkholz *et al.*, 2009 ▸). Evidence of intra­molecular π stacking between naphthyl and phenyl substituents is observed, generating close contacts ranging from 3.2 to 4.0 Å. Fig. 4 ▸ shows the distances between calculated centroids of two of the phospho­rus phenyl substituents and the nearest six membered carbon ring of a napthyl unit.

Compared to the Pd analogue (Véron *et al.*, 2013 ▸), the Pt—Cl bond lengths [Pt1—Cl1 = 2.3518 (8) Å; Pt1—Cl2 = 2.3536 (8) Å]) are only around 0.01 Å longer. The two Pt—Cl distances are also statistically indistinguishable, implying similar orbital overlap between the Pt^II^ metal centre and the strong *trans* phosphine donors. An only slightly acute Cl1—Pt1—Cl2 angle of 87.44 (3)° is observed, indicating slight steric repulsion from the di­phenyl­phosphine arms. Angles closer to the ideal of 90° are seen between *cis*-disposed phospho­rus and chlorine atoms. The bond lengths involving the Pt metal centre are similar to those in the enanti­opure (*R*-BINAP)PtCl_2_ (Doherty *et al.*, 2006 ▸); however, deviations are observed in several of the angles.

**Figure 5:**
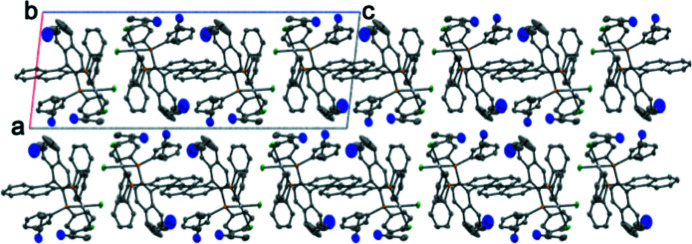
A projection showing the unit-cell contents and packing of (*rac*BINAP)PtCl_2_. Displacement ellipsoids are shown at 50% probability level. Hydrogen atoms are omitted for clarity.

In a single unit cell, four mol­ecules can be found (Fig. 5 ▸), with two of each enanti­omer present. Inter­estingly, no significant inter­molecular inter­actions are present within the sum of the van der Waals radii. The closest inter­molecular inter­action stems from hydrogen bonds between neighbouring aceto­nitrile solvent mol­ecules. These inter­actions are all greater than 3.40 Å and so were not investigated any further. Distances of 3.30 to 3.70 Å can be observed between naphthyl carbon atoms of neighbouring complexes; however, the arrangement is not stacked and so not likely to be significant.

## Synthesis and crystallization

Crystals of (*rac*BINAP)PtCl_2_ were obtained as a side-product from a reaction mixture of (COD)PtCl_2_ and a tridentate, di­aryl­amido-*N*,*N-*phenanthridine-based ligand (Mandapati *et al.*, 2019 ▸). BINAP was used to construct this ligand *via* a Pd-cross coupling reaction and was not completely removed from the proligand before metalation. Crystal-structure data were collected from a multi-faceted crystal of suitable size and quality selected from a representative sample of crystals of the same habit using an optical microscope.

## Refinement

Crystal data, data collection and structure refinement details are summarized in Table 2 ▸.

## Supplementary Material

Crystal structure: contains datablock(s) I. DOI: 10.1107/S2414314620010482/zq2254sup1.cif

Structure factors: contains datablock(s) I. DOI: 10.1107/S2414314620010482/zq2254Isup2.hkl

Supporting information file. DOI: 10.1107/S2414314620010482/zq2254Isup3.cml

Supporting tables and experimental information. DOI: 10.1107/S2414314620010482/zq2254sup4.pdf

CCDC reference: 2020002

Additional supporting information:  crystallographic information; 3D view; checkCIF report

## Figures and Tables

**Table 1:** Selected geometric parameters (Å, °)

Pt1—Cl1	2.3518 (8)	P1—Pt1	2.2447 (8)
Pt1—Cl2	2.3536 (8)	P2—Pt1	2.2422 (8)
			
Cl1—Pt1—Cl2	87.44 (3)	P2—Pt1—Cl1	170.91 (3)
P1—Pt1—Cl1	90.31 (3)	P2—Pt1—Cl2	90.62 (3)
P1—Pt1—Cl2	171.33 (3)	P2—Pt1—P1	92.87 (3)

**Table 2:** Experimental details

Crystal data	
Chemical formula	[PtCl_2_(C_44_H_32_P_2_)]·3C_2_H_3_N
*M*_r_	1011.79
Crystal system, space group	Monoclinic, *P*2_1_/*c*
Temperature (K)	150
*a*, *b*, *c* (Å)	11.3681 (4), 12.5001 (4), 30.7944 (11)
β (°)	96.439 (2)
*V* (Å^3^)	4348.4 (3)
*Z*	4
Radiation type	Mo *K*α
μ (mm^−1^)	3.46
Crystal size (mm)	0.39 × 0.19 × 0.13

Data collection	
Diffractometer	Bruker D8 Quest ECO CMOS
Absorption correction	Multi-scan (*SADABS*; Bruker, 2016 ▸)
*T*_min_, *T*_max_	0.553, 0.746
No. of measured, independent and observed [*I* > 2σ(*I*)] reflections	142092, 13302, 11205
*R*_int_	0.079
(sin θ/λ)_max_ (Å^−1^)	0.715

Refinement	
*R*[*F* ^2^ > 2σ(*F* ^2^)], *wR*(*F* ^2^), *S*	0.036, 0.072, 1.09
No. of reflections	13302
No. of parameters	526
No. of restraints	18
H-atom treatment	H-atom parameters constrained
Δρ_max_, Δρ_min_ (e Å^−3^)	1.25, −1.49

Computer programs: *APEX3* and *SAINT* (Bruker, 2016 ▸), *SHELXT* (Sheldrick, 2015*a* ▸), *SHELXL* (Sheldrick, 2015*b* ▸), *OLEX2* (Dolomanov *et al.*, 2009 ▸) and *PLATON* (Spek, 2020 ▸).

## full crystallographic data

### Crystal data

**Table:**

[PtCl_2_(C_44_H_32_P_2_)]·3C_2_H_3_N	*F*(000) = 2016
*M**_r_* = 1011.79	*D*_x_ = 1.546 Mg m^−^^3^
Monoclinic, *P*2_1_/*c*	Mo *K*α radiation, λ = 0.71073 Å
*a* = 11.3681 (4) Å	Cell parameters from 9175 reflections
*b* = 12.5001 (4) Å	θ = 2.4–30.5°
*c* = 30.7944 (11) Å	µ = 3.46 mm^−^^1^
β = 96.439 (2)°	*T* = 150 K
*V* = 4348.4 (3) Å^3^	Block, orange
*Z* = 4	0.39 × 0.19 × 0.13 mm

### Data collection

**Table:**

Bruker D8 Quest ECO CMOS diffractometer	11205 reflections with *I* > 2σ(*I*)
Radiation source: fine–focus tube	*R*_int_ = 0.079
φ and ω scans	θ_max_ = 30.6°, θ_min_ = 2.4°
Absorption correction: multi-scan (SADABS; Bruker, 2016)	*h* = −16→16
*T*_min_ = 0.553, *T*_max_ = 0.746	*k* = −17→17
142092 measured reflections	*l* = −44→44
13302 independent reflections	

### Refinement

**Table:**

Refinement on *F*^2^	Primary atom site location: dual
Least-squares matrix: full	Hydrogen site location: inferred from neighbouring sites
*R*[*F*^2^ > 2σ(*F*^2^)] = 0.036	H-atom parameters constrained
*wR*(*F*^2^) = 0.072	*w* = 1/[σ^2^(*F*_o_^2^) + (0.0143*P*)^2^ + 16.231*P*] where *P* = (*F*_o_^2^ + 2*F*_c_^2^)/3
*S* = 1.09	(Δ/σ)_max_ = 0.003
13302 reflections	Δρ_max_ = 1.25 e Å^−^^3^
526 parameters	Δρ_min_ = −1.49 e Å^−^^3^
18 restraints	

### Special details

**Table:**

Geometry. All esds (except the esd in the dihedral angle between two l.s. planes) are estimated using the full covariance matrix. The cell esds are taken into account individually in the estimation of esds in distances, angles and torsion angles; correlations between esds in cell parameters are only used when they are defined by crystal symmetry. An approximate (isotropic) treatment of cell esds is used for estimating esds involving l.s. planes.
Refinement. A single-crystal was mounted on a MiTiGen loop and data collection was carried out in a cold stream of nitrogen. All diffractometer manipulations were carried out using Bruker *APEX3* software (Bruker-AXS, 2016). Structure solution and refinement were carried out in the OLEX2 (Dolomanov *et al.*, 2009) program using *SHELXT* (Sheldrick, 2015*a*) and *SHELXL* (Sheldrick, 2015*b*) softwares. All hydrogen atoms within the structure were placed in geometrically idealized positions and were constrained to ride on their parent atoms (C–H = 0.95 Å). The absence of additional symmetry was confirmed using ADDSYM incorporated in the *PLATON* program (Spek, 2020). The presence of inter- or intramolecular hydrogen bonds was probed, but not observed below a limit of 3.40 Å with a D–H···A angle of less than 120°.

### Fractional atomic coordinates and isotropic or equivalent isotropic displacement parameters (Å^2^)

**Table:**

	*x*	*y*	*z*	*U*_iso_*/*U*_eq_	
C1	0.2521 (3)	0.1586 (3)	0.40291 (10)	0.0175 (6)	
C2	0.2621 (3)	0.0859 (3)	0.43763 (11)	0.0218 (7)	
H2	0.302614	0.020146	0.435151	0.026*	
C3	0.2128 (3)	0.1098 (4)	0.47570 (12)	0.0309 (9)	
H3	0.220148	0.060652	0.499378	0.037*	
C4	0.1533 (3)	0.2046 (4)	0.47912 (13)	0.0367 (10)	
H4	0.119892	0.220626	0.505285	0.044*	
C5	0.1415 (3)	0.2765 (4)	0.44510 (14)	0.0350 (9)	
H5	0.100082	0.341684	0.447939	0.042*	
C6	0.1902 (3)	0.2542 (3)	0.40638 (12)	0.0237 (7)	
H6	0.181422	0.303483	0.382742	0.028*	
C7	0.2479 (3)	0.0151 (3)	0.32864 (10)	0.0159 (6)	
C8	0.1587 (3)	−0.0380 (3)	0.34728 (12)	0.0231 (7)	
H8	0.135936	−0.014840	0.374495	0.028*	
C9	0.1026 (4)	−0.1259 (3)	0.32576 (13)	0.0305 (8)	
H9	0.041022	−0.161847	0.338345	0.037*	
C10	0.1359 (3)	−0.1609 (3)	0.28637 (13)	0.0272 (8)	
H10	0.098355	−0.221397	0.272208	0.033*	
C11	0.2243 (3)	−0.1073 (3)	0.26758 (11)	0.0228 (7)	
H11	0.247682	−0.131209	0.240550	0.027*	
C12	0.2784 (3)	−0.0190 (3)	0.28826 (10)	0.0182 (6)	
H12	0.337044	0.018842	0.274784	0.022*	
C13	0.4718 (3)	0.0833 (2)	0.37430 (9)	0.0123 (5)	
C14	0.5161 (3)	−0.0090 (2)	0.35467 (10)	0.0139 (6)	
H14	0.464981	−0.048898	0.334186	0.017*	
C15	0.6304 (3)	−0.0415 (2)	0.36460 (10)	0.0161 (6)	
H15	0.657915	−0.102753	0.350507	0.019*	
C16	0.7085 (3)	0.0145 (2)	0.39555 (10)	0.0147 (6)	
C17	0.8273 (3)	−0.0188 (3)	0.40605 (11)	0.0215 (7)	
H17	0.855246	−0.080496	0.392353	0.026*	
C18	0.9025 (3)	0.0369 (3)	0.43572 (12)	0.0247 (7)	
H18	0.981974	0.013862	0.442746	0.030*	
C19	0.8609 (3)	0.1288 (3)	0.45579 (11)	0.0232 (7)	
H19	0.913246	0.167801	0.476178	0.028*	
C20	0.7463 (3)	0.1628 (3)	0.44639 (10)	0.0176 (6)	
H20	0.720221	0.224811	0.460328	0.021*	
C21	0.6662 (3)	0.1061 (2)	0.41602 (10)	0.0140 (6)	
C22	0.5464 (3)	0.1407 (2)	0.40469 (9)	0.0123 (5)	
C23	0.5052 (3)	0.2397 (2)	0.42572 (9)	0.0131 (5)	
C24	0.4825 (3)	0.3333 (2)	0.40198 (9)	0.0128 (5)	
C25	0.4400 (3)	0.4247 (2)	0.42271 (10)	0.0158 (6)	
H25	0.426277	0.489099	0.406547	0.019*	
C26	0.4186 (3)	0.4219 (3)	0.46534 (10)	0.0171 (6)	
H26	0.387404	0.483429	0.478066	0.020*	
C27	0.4419 (3)	0.3292 (2)	0.49086 (10)	0.0148 (6)	
C28	0.4214 (3)	0.3262 (3)	0.53539 (10)	0.0195 (6)	
H28	0.391311	0.387923	0.548380	0.023*	
C29	0.4443 (3)	0.2361 (3)	0.55992 (10)	0.0219 (6)	
H29	0.429616	0.234811	0.589678	0.026*	
C30	0.4899 (3)	0.1448 (3)	0.54072 (10)	0.0199 (6)	
H30	0.505993	0.082119	0.557782	0.024*	
C31	0.5114 (3)	0.1453 (2)	0.49788 (10)	0.0162 (6)	
H31	0.542657	0.083009	0.485726	0.019*	
C32	0.4877 (3)	0.2373 (2)	0.47126 (9)	0.0128 (5)	
C33	0.4989 (3)	0.4806 (3)	0.33082 (10)	0.0168 (6)	
C34	0.3962 (3)	0.5418 (3)	0.33043 (11)	0.0203 (7)	
H34	0.325124	0.509001	0.337272	0.024*	
C35	0.3965 (4)	0.6497 (3)	0.32019 (11)	0.0252 (7)	
H35	0.326574	0.691091	0.320763	0.030*	
C36	0.4990 (4)	0.6970 (3)	0.30914 (12)	0.0307 (9)	
H36	0.499050	0.770707	0.301582	0.037*	
C37	0.6009 (4)	0.6378 (3)	0.30905 (13)	0.0302 (8)	
H37	0.671299	0.670836	0.301692	0.036*	
C38	0.6014 (3)	0.5294 (3)	0.31972 (12)	0.0236 (7)	
H38	0.671870	0.488684	0.319405	0.028*	
C39	0.6310 (3)	0.2806 (2)	0.33394 (10)	0.0154 (6)	
C40	0.6356 (3)	0.2005 (3)	0.30250 (10)	0.0174 (6)	
H40	0.565041	0.177771	0.285560	0.021*	
C41	0.7432 (3)	0.1544 (3)	0.29603 (12)	0.0245 (7)	
H41	0.745961	0.099678	0.274747	0.029*	
C42	0.8462 (3)	0.1873 (3)	0.32027 (13)	0.0265 (8)	
H42	0.919463	0.154959	0.315777	0.032*	
C43	0.8429 (3)	0.2678 (3)	0.35128 (12)	0.0257 (7)	
H43	0.914178	0.291634	0.367440	0.031*	
C44	0.7358 (3)	0.3132 (3)	0.35860 (11)	0.0207 (7)	
H44	0.733391	0.366573	0.380423	0.025*	
C45	0.0714 (4)	0.5037 (4)	0.32714 (18)	0.0429 (11)	
C46	0.0589 (4)	0.5036 (4)	0.28013 (17)	0.0473 (12)	
H46A	0.122395	0.546344	0.269788	0.071*	
H46B	−0.017963	0.534298	0.269059	0.071*	
H46C	0.063717	0.429940	0.269533	0.071*	
C47	0.8616 (4)	0.2521 (4)	0.56830 (15)	0.0408 (10)	
C48	0.7545 (5)	0.3114 (6)	0.5712 (2)	0.0727 (19)	
H48A	0.774248	0.385073	0.580191	0.109*	
H48B	0.709346	0.277725	0.592839	0.109*	
H48C	0.706889	0.311867	0.542659	0.109*	
Cl1	0.14268 (7)	0.21270 (7)	0.27128 (3)	0.02745 (19)	
Cl2	0.34019 (8)	0.38528 (6)	0.24790 (2)	0.02091 (16)	
N1	0.0830 (6)	0.5016 (5)	0.3642 (2)	0.0805 (17)	
N2	0.9465 (5)	0.2073 (4)	0.56653 (16)	0.0643 (14)	
P1	0.32310 (7)	0.13146 (6)	0.35451 (2)	0.01190 (14)	
P2	0.49025 (7)	0.33817 (6)	0.34282 (2)	0.01222 (14)	
Pt1	0.32836 (2)	0.26525 (2)	0.30614 (2)	0.01414 (3)	
C51	0.8660 (10)	0.5704 (9)	0.4281 (4)	0.144 (3)	
H51A	0.894116	0.636153	0.443150	0.216*	
H51B	0.790640	0.584487	0.410230	0.216*	
H51C	0.924592	0.546071	0.409218	0.216*	
C52	0.8493 (11)	0.4896 (11)	0.4595 (5)	0.142 (3)	
N3	0.8066 (10)	0.4370 (8)	0.4778 (4)	0.144 (3)	

### Atomic displacement parameters (Å^2^)

**Table:**

	*U*^11^	*U*^22^	*U*^33^	*U*^12^	*U*^13^	*U*^23^
C1	0.0155 (14)	0.0225 (16)	0.0150 (14)	−0.0041 (12)	0.0049 (11)	−0.0059 (12)
C2	0.0181 (16)	0.0295 (18)	0.0180 (15)	−0.0067 (13)	0.0028 (12)	0.0005 (13)
C3	0.0261 (19)	0.053 (3)	0.0149 (16)	−0.0179 (17)	0.0061 (14)	−0.0020 (15)
C4	0.0241 (18)	0.066 (3)	0.0225 (18)	−0.0163 (19)	0.0125 (15)	−0.0214 (18)
C5	0.0227 (18)	0.045 (2)	0.039 (2)	−0.0012 (17)	0.0096 (16)	−0.0223 (19)
C6	0.0174 (15)	0.0272 (19)	0.0268 (17)	−0.0012 (13)	0.0036 (12)	−0.0075 (14)
C7	0.0163 (14)	0.0158 (15)	0.0148 (14)	−0.0016 (11)	−0.0016 (11)	0.0010 (11)
C8	0.0231 (17)	0.0245 (18)	0.0220 (16)	−0.0072 (13)	0.0038 (13)	−0.0031 (13)
C9	0.031 (2)	0.028 (2)	0.032 (2)	−0.0154 (16)	0.0050 (16)	−0.0027 (15)
C10	0.0298 (19)	0.0165 (16)	0.034 (2)	−0.0084 (14)	−0.0038 (15)	−0.0042 (14)
C11	0.0272 (18)	0.0200 (16)	0.0202 (16)	−0.0010 (13)	−0.0010 (13)	−0.0050 (13)
C12	0.0213 (16)	0.0164 (15)	0.0169 (15)	0.0004 (12)	0.0023 (12)	−0.0017 (11)
C13	0.0143 (13)	0.0108 (13)	0.0122 (13)	−0.0014 (10)	0.0030 (10)	0.0018 (10)
C14	0.0184 (15)	0.0116 (13)	0.0116 (13)	−0.0014 (11)	0.0017 (11)	−0.0012 (10)
C15	0.0203 (15)	0.0139 (14)	0.0147 (14)	0.0022 (11)	0.0042 (11)	−0.0017 (11)
C16	0.0140 (14)	0.0157 (14)	0.0152 (14)	0.0018 (11)	0.0045 (11)	0.0007 (11)
C17	0.0186 (16)	0.0241 (17)	0.0222 (16)	0.0047 (13)	0.0035 (13)	−0.0016 (13)
C18	0.0121 (15)	0.035 (2)	0.0271 (18)	0.0024 (13)	0.0014 (13)	0.0002 (15)
C19	0.0176 (16)	0.0303 (19)	0.0211 (16)	−0.0035 (13)	−0.0002 (13)	−0.0020 (13)
C20	0.0150 (14)	0.0200 (15)	0.0182 (15)	−0.0007 (12)	0.0032 (11)	−0.0015 (12)
C21	0.0138 (14)	0.0150 (14)	0.0134 (13)	−0.0017 (11)	0.0031 (11)	0.0010 (11)
C22	0.0153 (14)	0.0098 (13)	0.0124 (13)	−0.0026 (10)	0.0038 (10)	0.0004 (10)
C23	0.0143 (13)	0.0111 (13)	0.0141 (13)	−0.0011 (11)	0.0020 (10)	−0.0004 (10)
C24	0.0126 (13)	0.0125 (13)	0.0129 (13)	−0.0020 (10)	0.0005 (10)	−0.0012 (10)
C25	0.0176 (15)	0.0136 (14)	0.0159 (14)	0.0016 (11)	0.0008 (11)	0.0012 (11)
C26	0.0212 (16)	0.0140 (14)	0.0162 (14)	0.0022 (12)	0.0031 (12)	−0.0026 (11)
C27	0.0158 (14)	0.0146 (14)	0.0143 (14)	−0.0012 (11)	0.0034 (11)	−0.0025 (11)
C28	0.0240 (17)	0.0196 (16)	0.0161 (15)	−0.0012 (13)	0.0068 (12)	−0.0045 (12)
C29	0.0301 (17)	0.0230 (16)	0.0136 (13)	−0.0032 (14)	0.0061 (12)	−0.0017 (12)
C30	0.0286 (18)	0.0169 (15)	0.0144 (14)	−0.0003 (13)	0.0032 (13)	0.0026 (11)
C31	0.0195 (15)	0.0138 (14)	0.0154 (14)	−0.0007 (11)	0.0020 (11)	0.0002 (11)
C32	0.0135 (12)	0.0119 (13)	0.0129 (12)	−0.0012 (11)	0.0010 (10)	−0.0007 (10)
C33	0.0250 (16)	0.0147 (14)	0.0105 (13)	−0.0017 (12)	0.0009 (12)	0.0013 (10)
C34	0.0293 (18)	0.0139 (15)	0.0175 (15)	0.0008 (13)	0.0015 (13)	0.0026 (11)
C35	0.040 (2)	0.0167 (16)	0.0188 (16)	0.0063 (14)	0.0027 (14)	0.0001 (12)
C36	0.059 (3)	0.0129 (15)	0.0209 (17)	−0.0026 (16)	0.0075 (17)	0.0009 (13)
C37	0.043 (2)	0.0193 (17)	0.0300 (19)	−0.0100 (16)	0.0134 (17)	0.0004 (14)
C38	0.0307 (19)	0.0180 (16)	0.0238 (17)	−0.0045 (14)	0.0108 (14)	0.0023 (13)
C39	0.0167 (14)	0.0157 (15)	0.0142 (13)	−0.0010 (11)	0.0037 (11)	0.0025 (11)
C40	0.0206 (15)	0.0154 (14)	0.0172 (14)	−0.0013 (12)	0.0058 (12)	0.0018 (11)
C41	0.0281 (18)	0.0193 (17)	0.0284 (18)	0.0025 (14)	0.0134 (15)	0.0000 (13)
C42	0.0178 (16)	0.0291 (19)	0.0342 (19)	0.0051 (14)	0.0097 (14)	0.0091 (15)
C43	0.0167 (15)	0.0324 (18)	0.0283 (17)	−0.0045 (14)	0.0034 (13)	0.0076 (15)
C44	0.0190 (16)	0.0237 (17)	0.0195 (15)	−0.0051 (13)	0.0025 (12)	0.0009 (13)
C45	0.030 (2)	0.043 (3)	0.056 (3)	0.0023 (19)	0.006 (2)	−0.003 (2)
C46	0.036 (2)	0.048 (3)	0.054 (3)	0.010 (2)	−0.007 (2)	−0.002 (2)
C47	0.046 (3)	0.043 (3)	0.034 (2)	0.003 (2)	0.0074 (18)	−0.0024 (19)
C48	0.037 (3)	0.092 (5)	0.088 (5)	0.011 (3)	0.001 (3)	−0.031 (4)
Cl1	0.0199 (4)	0.0220 (4)	0.0371 (5)	−0.0016 (3)	−0.0112 (3)	0.0027 (3)
Cl2	0.0344 (4)	0.0152 (3)	0.0128 (3)	0.0019 (3)	0.0008 (3)	0.0034 (3)
N1	0.091 (5)	0.086 (4)	0.066 (4)	0.013 (3)	0.019 (3)	−0.001 (3)
N2	0.071 (3)	0.069 (3)	0.054 (3)	0.029 (3)	0.013 (2)	0.001 (2)
P1	0.0128 (3)	0.0113 (3)	0.0118 (3)	−0.0015 (3)	0.0020 (3)	−0.0003 (3)
P2	0.0151 (4)	0.0104 (3)	0.0114 (3)	−0.0009 (3)	0.0024 (3)	0.0009 (3)
Pt1	0.01585 (5)	0.01254 (5)	0.01380 (5)	0.00102 (5)	0.00066 (4)	0.00026 (4)
C51	0.144 (6)	0.130 (7)	0.170 (8)	−0.018 (5)	0.072 (4)	−0.085 (6)
C52	0.142 (6)	0.128 (7)	0.168 (8)	−0.018 (5)	0.071 (5)	−0.086 (6)
N3	0.145 (6)	0.130 (7)	0.170 (8)	−0.016 (5)	0.068 (4)	−0.085 (6)

### Geometric parameters (Å, º)

**Table:**

C1—C2	1.398 (5)	C27—C32	1.423 (4)
C1—C6	1.397 (5)	C28—H28	0.9500
C1—P1	1.805 (3)	C28—C29	1.366 (5)
C2—H2	0.9500	C29—H29	0.9500
C2—C3	1.387 (5)	C29—C30	1.410 (5)
C3—H3	0.9500	C30—H30	0.9500
C3—C4	1.374 (6)	C30—C31	1.368 (4)
C4—H4	0.9500	C31—H31	0.9500
C4—C5	1.376 (7)	C31—C32	1.421 (4)
C5—H5	0.9500	C33—C34	1.395 (5)
C5—C6	1.398 (5)	C33—C38	1.391 (5)
C6—H6	0.9500	C33—P2	1.824 (3)
C7—C8	1.389 (5)	C34—H34	0.9500
C7—C12	1.395 (4)	C34—C35	1.386 (5)
C7—P1	1.825 (3)	C35—H35	0.9500
C8—H8	0.9500	C35—C36	1.383 (6)
C8—C9	1.399 (5)	C36—H36	0.9500
C9—H9	0.9500	C36—C37	1.375 (6)
C9—C10	1.381 (5)	C37—H37	0.9500
C10—H10	0.9500	C37—C38	1.394 (5)
C10—C11	1.387 (5)	C38—H38	0.9500
C11—H11	0.9500	C39—C40	1.397 (4)
C11—C12	1.383 (5)	C39—C44	1.399 (4)
C12—H12	0.9500	C39—P2	1.803 (3)
C13—C14	1.421 (4)	C40—H40	0.9500
C13—C22	1.390 (4)	C40—C41	1.387 (5)
C13—P1	1.833 (3)	C41—H41	0.9500
C14—H14	0.9500	C41—C42	1.379 (5)
C14—C15	1.363 (4)	C42—H42	0.9500
C15—H15	0.9500	C42—C43	1.390 (6)
C15—C16	1.413 (4)	C43—H43	0.9500
C16—C17	1.416 (4)	C43—C44	1.385 (5)
C16—C21	1.418 (4)	C44—H44	0.9500
C17—H17	0.9500	C45—C46	1.438 (7)
C17—C18	1.369 (5)	C45—N1	1.134 (7)
C18—H18	0.9500	C46—H46A	0.9800
C18—C19	1.411 (5)	C46—H46B	0.9800
C19—H19	0.9500	C46—H46C	0.9800
C19—C20	1.369 (5)	C47—C48	1.437 (7)
C20—H20	0.9500	C47—N2	1.123 (6)
C20—C21	1.419 (4)	C48—H48A	0.9800
C21—C22	1.434 (4)	C48—H48B	0.9800
C22—C23	1.497 (4)	C48—H48C	0.9800
C23—C24	1.389 (4)	Pt1—Cl1	2.3518 (8)
C23—C32	1.439 (4)	Pt1—Cl2	2.3536 (8)
C24—C25	1.420 (4)	P1—Pt1	2.2447 (8)
C24—P2	1.835 (3)	P2—Pt1	2.2422 (8)
C25—H25	0.9500	C51—H51A	0.9800
C25—C26	1.362 (4)	C51—H51B	0.9800
C26—H26	0.9500	C51—H51C	0.9800
C26—C27	1.408 (4)	C51—C52	1.426 (17)
C27—C28	1.417 (4)	C52—N3	1.023 (15)
			
C2—C1—P1	120.3 (3)	C30—C29—H29	120.3
C6—C1—C2	119.7 (3)	C29—C30—H30	119.5
C6—C1—P1	120.0 (3)	C31—C30—C29	121.0 (3)
C1—C2—H2	120.0	C31—C30—H30	119.5
C3—C2—C1	120.1 (4)	C30—C31—H31	119.4
C3—C2—H2	120.0	C30—C31—C32	121.1 (3)
C2—C3—H3	120.0	C32—C31—H31	119.4
C4—C3—C2	119.9 (4)	C27—C32—C23	119.6 (3)
C4—C3—H3	120.0	C31—C32—C23	122.8 (3)
C3—C4—H4	119.6	C31—C32—C27	117.6 (3)
C3—C4—C5	120.8 (3)	C34—C33—P2	118.2 (2)
C5—C4—H4	119.6	C38—C33—C34	118.8 (3)
C4—C5—H5	119.8	C38—C33—P2	122.9 (3)
C4—C5—C6	120.4 (4)	C33—C34—H34	119.6
C6—C5—H5	119.8	C35—C34—C33	120.8 (3)
C1—C6—C5	119.1 (4)	C35—C34—H34	119.6
C1—C6—H6	120.4	C34—C35—H35	120.1
C5—C6—H6	120.4	C36—C35—C34	119.7 (4)
C8—C7—C12	119.2 (3)	C36—C35—H35	120.1
C8—C7—P1	121.9 (3)	C35—C36—H36	119.9
C12—C7—P1	118.8 (2)	C37—C36—C35	120.2 (3)
C7—C8—H8	120.2	C37—C36—H36	119.9
C7—C8—C9	119.7 (3)	C36—C37—H37	119.9
C9—C8—H8	120.2	C36—C37—C38	120.2 (4)
C8—C9—H9	119.7	C38—C37—H37	119.9
C10—C9—C8	120.6 (3)	C33—C38—C37	120.2 (4)
C10—C9—H9	119.7	C33—C38—H38	119.9
C9—C10—H10	120.1	C37—C38—H38	119.9
C9—C10—C11	119.7 (3)	C40—C39—C44	119.3 (3)
C11—C10—H10	120.1	C40—C39—P2	119.6 (2)
C10—C11—H11	120.0	C44—C39—P2	121.1 (2)
C12—C11—C10	119.9 (3)	C39—C40—H40	120.0
C12—C11—H11	120.0	C41—C40—C39	120.0 (3)
C7—C12—H12	119.6	C41—C40—H40	120.0
C11—C12—C7	120.8 (3)	C40—C41—H41	119.8
C11—C12—H12	119.6	C42—C41—C40	120.5 (3)
C14—C13—P1	118.9 (2)	C42—C41—H41	119.8
C22—C13—C14	119.1 (3)	C41—C42—H42	120.0
C22—C13—P1	121.7 (2)	C41—C42—C43	120.1 (3)
C13—C14—H14	119.3	C43—C42—H42	120.0
C15—C14—C13	121.4 (3)	C42—C43—H43	120.0
C15—C14—H14	119.3	C44—C43—C42	120.0 (3)
C14—C15—H15	119.5	C44—C43—H43	120.0
C14—C15—C16	121.0 (3)	C39—C44—H44	119.9
C16—C15—H15	119.5	C43—C44—C39	120.2 (3)
C15—C16—C17	121.3 (3)	C43—C44—H44	119.9
C15—C16—C21	118.8 (3)	N1—C45—C46	178.3 (6)
C17—C16—C21	119.9 (3)	C45—C46—H46A	109.5
C16—C17—H17	119.6	C45—C46—H46B	109.5
C18—C17—C16	120.7 (3)	C45—C46—H46C	109.5
C18—C17—H17	119.6	H46A—C46—H46B	109.5
C17—C18—H18	120.3	H46A—C46—H46C	109.5
C17—C18—C19	119.5 (3)	H46B—C46—H46C	109.5
C19—C18—H18	120.3	N2—C47—C48	178.6 (6)
C18—C19—H19	119.4	C47—C48—H48A	109.5
C20—C19—C18	121.2 (3)	C47—C48—H48B	109.5
C20—C19—H19	119.4	C47—C48—H48C	109.5
C19—C20—H20	119.7	H48A—C48—H48B	109.5
C19—C20—C21	120.6 (3)	H48A—C48—H48C	109.5
C21—C20—H20	119.7	H48B—C48—H48C	109.5
C16—C21—C20	118.2 (3)	C1—P1—C7	106.21 (15)
C16—C21—C22	119.7 (3)	C1—P1—C13	105.57 (14)
C20—C21—C22	122.1 (3)	C1—P1—Pt1	117.11 (12)
C13—C22—C21	120.0 (3)	C7—P1—C13	104.64 (14)
C13—C22—C23	121.4 (3)	C7—P1—Pt1	110.51 (10)
C21—C22—C23	118.6 (3)	C13—P1—Pt1	111.90 (10)
C24—C23—C22	121.2 (3)	C24—P2—Pt1	111.01 (10)
C24—C23—C32	119.5 (3)	C33—P2—C24	104.08 (14)
C32—C23—C22	119.3 (3)	C33—P2—Pt1	110.78 (11)
C23—C24—C25	119.6 (3)	C39—P2—C24	106.24 (14)
C23—C24—P2	121.6 (2)	C39—P2—C33	106.87 (15)
C25—C24—P2	118.5 (2)	C39—P2—Pt1	116.96 (11)
C24—C25—H25	119.4	Cl1—Pt1—Cl2	87.44 (3)
C26—C25—C24	121.3 (3)	P1—Pt1—Cl1	90.31 (3)
C26—C25—H25	119.4	P1—Pt1—Cl2	171.33 (3)
C25—C26—H26	119.5	P2—Pt1—Cl1	170.91 (3)
C25—C26—C27	121.1 (3)	P2—Pt1—Cl2	90.62 (3)
C27—C26—H26	119.5	P2—Pt1—P1	92.87 (3)
C26—C27—C28	121.4 (3)	H51A—C51—H51B	109.5
C26—C27—C32	118.8 (3)	H51A—C51—H51C	109.5
C28—C27—C32	119.8 (3)	H51B—C51—H51C	109.5
C27—C28—H28	119.5	C52—C51—H51A	109.5
C29—C28—C27	121.0 (3)	C52—C51—H51B	109.5
C29—C28—H28	119.5	C52—C51—H51C	109.5
C28—C29—H29	120.3	N3—C52—C51	159.4 (19)
C28—C29—C30	119.5 (3)		
			
C1—C2—C3—C4	0.5 (5)	C23—C24—P2—C33	−163.2 (3)
C2—C1—C6—C5	1.4 (5)	C23—C24—P2—C39	−50.6 (3)
C2—C1—P1—C7	66.5 (3)	C23—C24—P2—Pt1	77.5 (3)
C2—C1—P1—C13	−44.3 (3)	C24—C23—C32—C27	3.0 (4)
C2—C1—P1—Pt1	−169.6 (2)	C24—C23—C32—C31	−178.7 (3)
C2—C3—C4—C5	0.2 (6)	C24—C25—C26—C27	2.3 (5)
C3—C4—C5—C6	−0.1 (6)	C25—C24—P2—C33	23.5 (3)
C4—C5—C6—C1	−0.7 (5)	C25—C24—P2—C39	136.1 (2)
C6—C1—C2—C3	−1.3 (5)	C25—C24—P2—Pt1	−95.7 (2)
C6—C1—P1—C7	−115.7 (3)	C25—C26—C27—C28	179.1 (3)
C6—C1—P1—C13	133.5 (3)	C25—C26—C27—C32	−0.5 (5)
C6—C1—P1—Pt1	8.2 (3)	C26—C27—C28—C29	−180.0 (3)
C7—C8—C9—C10	0.6 (6)	C26—C27—C32—C23	−2.2 (4)
C8—C7—C12—C11	−2.6 (5)	C26—C27—C32—C31	179.4 (3)
C8—C7—P1—C1	−2.9 (3)	C27—C28—C29—C30	0.5 (5)
C8—C7—P1—C13	108.5 (3)	C28—C27—C32—C23	178.3 (3)
C8—C7—P1—Pt1	−130.9 (3)	C28—C27—C32—C31	−0.1 (4)
C8—C9—C10—C11	−1.1 (6)	C28—C29—C30—C31	−0.1 (5)
C9—C10—C11—C12	−0.2 (6)	C29—C30—C31—C32	−0.4 (5)
C10—C11—C12—C7	2.1 (5)	C30—C31—C32—C23	−177.8 (3)
C12—C7—C8—C9	1.2 (5)	C30—C31—C32—C27	0.5 (5)
C12—C7—P1—C1	174.9 (3)	C32—C23—C24—C25	−1.3 (4)
C12—C7—P1—C13	−73.7 (3)	C32—C23—C24—P2	−174.5 (2)
C12—C7—P1—Pt1	46.9 (3)	C32—C27—C28—C29	−0.4 (5)
C13—C14—C15—C16	1.3 (5)	C33—C34—C35—C36	1.6 (5)
C13—C22—C23—C24	−69.7 (4)	C34—C33—C38—C37	0.9 (5)
C13—C22—C23—C32	109.7 (3)	C34—C33—P2—C24	−75.0 (3)
C14—C13—C22—C21	−0.3 (4)	C34—C33—P2—C39	172.9 (2)
C14—C13—C22—C23	178.8 (3)	C34—C33—P2—Pt1	44.4 (3)
C14—C13—P1—C1	134.1 (2)	C34—C35—C36—C37	−1.2 (6)
C14—C13—P1—C7	22.2 (3)	C35—C36—C37—C38	0.6 (6)
C14—C13—P1—Pt1	−97.5 (2)	C36—C37—C38—C33	−0.4 (6)
C14—C15—C16—C17	180.0 (3)	C38—C33—C34—C35	−1.5 (5)
C14—C15—C16—C21	−0.5 (5)	C38—C33—P2—C24	108.7 (3)
C15—C16—C17—C18	179.2 (3)	C38—C33—P2—C39	−3.5 (3)
C15—C16—C21—C20	−178.8 (3)	C38—C33—P2—Pt1	−131.9 (3)
C15—C16—C21—C22	−0.7 (4)	C39—C40—C41—C42	−0.4 (5)
C16—C17—C18—C19	−0.4 (5)	C40—C39—C44—C43	1.2 (5)
C16—C21—C22—C13	1.0 (4)	C40—C39—P2—C24	129.9 (3)
C16—C21—C22—C23	−178.1 (3)	C40—C39—P2—C33	−119.4 (3)
C17—C16—C21—C20	0.7 (4)	C40—C39—P2—Pt1	5.4 (3)
C17—C16—C21—C22	178.9 (3)	C40—C41—C42—C43	−0.4 (5)
C17—C18—C19—C20	0.6 (6)	C41—C42—C43—C44	1.6 (5)
C18—C19—C20—C21	−0.1 (5)	C42—C43—C44—C39	−1.9 (5)
C19—C20—C21—C16	−0.6 (5)	C44—C39—C40—C41	0.0 (5)
C19—C20—C21—C22	−178.6 (3)	C44—C39—P2—C24	−48.8 (3)
C20—C21—C22—C13	179.1 (3)	C44—C39—P2—C33	61.9 (3)
C20—C21—C22—C23	0.0 (4)	C44—C39—P2—Pt1	−173.4 (2)
C21—C16—C17—C18	−0.3 (5)	P1—C1—C2—C3	176.5 (3)
C21—C22—C23—C24	109.4 (3)	P1—C1—C6—C5	−176.4 (3)
C21—C22—C23—C32	−71.3 (4)	P1—C7—C8—C9	179.0 (3)
C22—C13—C14—C15	−0.9 (4)	P1—C7—C12—C11	179.6 (3)
C22—C13—P1—C1	−52.6 (3)	P1—C13—C14—C15	172.6 (2)
C22—C13—P1—C7	−164.4 (2)	P1—C13—C22—C21	−173.6 (2)
C22—C13—P1—Pt1	75.9 (2)	P1—C13—C22—C23	5.5 (4)
C22—C23—C24—C25	178.1 (3)	P2—C24—C25—C26	172.0 (3)
C22—C23—C24—P2	4.9 (4)	P2—C33—C34—C35	−178.0 (3)
C22—C23—C32—C27	−176.3 (3)	P2—C33—C38—C37	177.2 (3)
C22—C23—C32—C31	2.0 (4)	P2—C39—C40—C41	−178.8 (2)
C23—C24—C25—C26	−1.4 (5)	P2—C39—C44—C43	179.9 (3)
