# Supplementary material for: [2,2′-Bis(di­phenyl­phosphan­yl)-1,1′-binaphthyl-κ2 P,P′]di­chlorido­platinum(II) acetonitrile trisolvate
Source: IUCrdata. 2020 Aug 4;5(Pt 8):x201048. doi: 10.1107/S2414314620010482 (PMC9462241; doi:10.1107/S2414314620010482)
Supplement: Supplementary file 4 [file x-05-x201048-sup4.pdf]

Supporting Information

for

Structure elucidation of racemic dichloro[2,2'-*bis*(diphenylphosphino)-1,1'-binaphthyl]platinum(II)

Jason D. Braun[a], Guneet Uppal[a] and David E. Herbert[a]*

[a] Department of Chemistry and the Manitoba Institute of Materials, University of Manitoba, Winnipeg, Manitoba, R3T 2N2, Canada

*david.herbert@umanitoba.ca

Abstract: The crystal structure (150 K) of racemic dichloro[2,2'-bis(diphenylphosphino)-1,1'-binaphthyl]platinum(II) (*rac*BINAP)$PtCl_2$ has been determined. The asymmetric unit consists of a single molecule of the title compound co-crystallized with three acetonitrile solvent molecules. Packing the unit cell confirms the racemic nature of the compound. Four molecules are observed in the unit cell, with *R* and *S* enantiomers present in a 2:2 ratio. Evidence of intramolecular π-stacking is observed within aromatic ligand motifs with no discernable intermolecular interactions.

Table S1. Crystal structure data

Identification code	deh217
Empirical formula	$C_{50}H_{41}Cl_2N_3P_2Pt$
Formula weight	1011.79
Temperature/K	150.0
Crystal system	monoclinic
Space group	$P2_1/c$
a/Å	11.3681(4)
b/Å	12.5001(4)
c/Å	30.7944(11)
α/°	90
β/°	96.439(2)
γ/°	90
Volume/$Å^3$	4348.4(3)
Z	4
$\rho_{calc}g/cm^3$	1.546
μ/mm^{-1}	3.462
F(000)	2016.0
Crystal size/mm^3	0.389 × 0.193 × 0.126
Radiation	MoKα (λ = 0.71073)
2Θ range for data collection/°	4.86 to 61.108
Index ranges	$-16 \le h \le 16, -17 \le k \le 17, -44 \le l \le 44$
Reflections collected	142092
Independent reflections	13302 [R_{int} = 0.0793, R_{sigma} = 0.0342]
Data/restraints/parameters	13302/0/526
Goodness-of-fit on F^2	1.088
Final R indexes [I>=2σ (I)]	R_1 = 0.0352, wR_2 = 0.0655
Final R indexes [all data]	R_1 = 0.0492, wR_2 = 0.0696
Largest diff. peak/hole / e $Å^{-3}$	1.23/-1.49

Table S2. Fractional Atomic Coordinates (×10^4) and Equivalent Isotropic Displacement Parameters (Å^2×10^3) for deh217. U_{eq} is defined as 1/3 of the trace of the orthogonalised U_{IJ} tensor.

Atom	*x*	*y*	*z*	U_{eq}
C1	2520(3)	1586(3)	4028.9(10)	17.4(6)
C2	2621(3)	859(3)	4376.3(11)	21.8(7)
C3	2128(3)	1098(4)	4756.7(11)	30.9(9)
C4	1533(3)	2046(4)	4791.3(12)	36.8(10)
C5	1415(3)	2765(4)	4451.0(13)	35.0(9)
C6	1902(3)	2542(3)	4063.8(11)	23.7(7)
C7	2479(3)	150(2)	3286.6(10)	16.0(6)
C8	1586(3)	-381(3)	3472.5(11)	23.0(7)
C9	1025(3)	-1259(3)	3257.6(13)	30.4(8)
C10	1359(3)	-1609(3)	2863.9(12)	27.2(8)
C11	2243(3)	-1073(3)	2676.2(11)	22.8(7)
C12	2784(3)	-190(3)	2882.5(10)	18.2(6)
C13	4718(3)	833(2)	3742.9(9)	12.3(5)
C14	5161(3)	-90(2)	3547.0(9)	13.9(5)
C15	6304(3)	-415(2)	3646.1(10)	16.1(6)
C16	7085(3)	145(2)	3955.3(10)	14.8(6)
C17	8273(3)	-188(3)	4060.5(11)	21.7(7)
C18	9024(3)	369(3)	4356.9(12)	24.8(7)
C19	8610(3)	1288(3)	4557.8(11)	23.2(7)
C20	7463(3)	1628(3)	4463.9(10)	17.6(6)
C21	6662(3)	1061(2)	4160.2(9)	14.0(5)
C22	5465(3)	1406(2)	4047.0(9)	12.3(5)
C23	5052(2)	2397(2)	4257.2(9)	13.2(5)
C24	4825(3)	3333(2)	4019.6(9)	12.8(5)
C25	4400(3)	4247(2)	4227.2(10)	15.7(6)
C26	4186(3)	4219(2)	4653.2(10)	17.0(6)
C27	4419(3)	3292(2)	4908.5(10)	14.8(6)
C28	4214(3)	3263(3)	5353.9(10)	19.5(6)
C29	4443(3)	2360(3)	5599.1(10)	21.8(6)
C30	4900(3)	1448(3)	5407.2(10)	20.0(6)
C31	5114(3)	1453(2)	4978.8(10)	16.3(6)
C32	4877(2)	2373(2)	4712.5(9)	12.8(5)

C33	4990(3)	4806(2)	3308.2(10)	16.8(6)
C34	3961(3)	5418(2)	3304.4(10)	20.3(6)
C35	3965(4)	6497(3)	3201.9(11)	25.2(7)
C36	4990(4)	6970(3)	3091.4(11)	30.7(8)
C37	6010(4)	6378(3)	3090.2(12)	30.1(8)
C38	6014(3)	5294(3)	3197.0(11)	23.6(7)
C39	6311(3)	2805(2)	3339.4(9)	15.3(6)
C40	6356(3)	2006(2)	3024.9(10)	17.4(6)
C41	7432(3)	1544(3)	2960.1(12)	24.4(7)
C42	8462(3)	1873(3)	3202.7(12)	26.5(7)
C43	8429(3)	2678(3)	3512.8(11)	25.7(7)
C44	7358(3)	3131(3)	3585.9(11)	20.7(6)
C45	9287(4)	10037(4)	1728.7(18)	43.0(10)
C46	9411(4)	10036(4)	2198.8(16)	47.3(12)
C47	8617(4)	2479(4)	683.0(14)	40.8(10)
C48	7544(5)	1886(6)	712(2)	72.7(19)
Cl1	1426.8(7)	2127.0(7)	2712.9(3)	27.44(18)
Cl2	3402.1(8)	3852.8(6)	2479.0(2)	20.89(15)
N1	9171(5)	10016(5)	1357.9(19)	80.2(16)
N2	9465(5)	2927(4)	665.2(15)	64.3(13)
P1	3231.1(7)	1314.6(6)	3545.1(2)	11.88(14)
P2	4902.6(7)	3381.7(6)	3428.2(2)	12.22(14)
Pt1	3283.6(2)	2652.5(2)	3061.4(2)	14.15(3)
C51	1308(12)	4269(10)	5722(5)	230(10)
C52	1577(9)	5052(9)	5431(4)	112(4)
N3	1931(11)	5641(8)	5223(3)	143(4)

Table S3. Anisotropic Displacement Parameters (Å^2×10^3) for deh217. The anisotropic displacement factor exponent takes the form: $-2\pi^2[h^2a^{*2}U_{11}+2hka^*b^*U_{12}+\ldots]$.

Atom	U_{11}	U_{22}	U_{33}	U_{23}	U_{13}	U_{12}
C1	15.4(14)	22.6(16)	15.0(14)	-5.9(11)	4.8(11)	-4.0(12)
C2	18.0(15)	29.6(18)	18.1(15)	0.4(13)	2.9(12)	-6.7(13)
C3	26.1(18)	53(2)	15.0(15)	-2.0(15)	6.0(13)	-17.7(17)
C4	24.0(18)	66(3)	22.5(18)	-21.4(18)	12.5(14)	-16.1(18)
C5	22.6(17)	45(2)	39(2)	-22.3(18)	9.7(15)	-1.3(16)
C6	17.4(14)	27.1(19)	26.8(16)	-7.4(13)	3.6(12)	-1.3(13)
C7	16.6(14)	15.5(14)	15.1(13)	0.9(11)	-1.8(11)	-1.9(11)
C8	23.2(17)	23.9(17)	22.2(16)	-3.1(13)	3.4(13)	-7.8(13)
C9	30.5(19)	28.5(19)	32.5(19)	-2.5(15)	4.9(15)	-15.2(15)
C10	29.9(19)	16.4(16)	33.7(19)	-4.3(14)	-3.9(15)	-8.3(13)
C11	27.3(17)	19.9(16)	20.4(15)	-5.1(12)	-1.2(13)	-0.8(13)
C12	21.4(16)	16.5(15)	16.8(14)	-1.7(11)	2.3(12)	0.3(12)
C13	14.4(13)	10.9(13)	12.1(12)	1.9(10)	3.1(10)	-1.3(10)
C14	18.5(14)	11.5(13)	11.6(13)	-1.2(10)	1.7(11)	-1.5(11)
C15	20.5(15)	13.8(14)	14.6(13)	-1.6(10)	4.4(11)	2.3(11)
C16	14.1(13)	15.8(14)	15.3(13)	0.8(11)	4.5(11)	1.6(11)
C17	19.1(16)	23.5(17)	22.8(16)	-1.6(12)	3.3(12)	4.6(12)
C18	12.3(15)	35(2)	27.2(17)	0.4(14)	1.0(13)	2.5(13)
C19	17.8(15)	30.1(18)	21.3(16)	-2.0(13)	0.3(12)	-3.8(13)
C20	15.0(14)	20.0(15)	18.2(14)	-1.6(11)	3.4(11)	-0.8(11)
C21	13.8(13)	15.1(14)	13.5(13)	1.2(10)	3.1(10)	-1.6(10)
C22	15.2(13)	9.7(12)	12.4(12)	0.5(10)	3.8(10)	-2.7(10)
C23	14.3(12)	11.0(12)	14.2(12)	-0.5(10)	2.0(10)	-1.2(10)
C24	12.6(13)	12.5(13)	12.9(13)	-1.3(10)	0.4(10)	-1.9(10)
C25	17.7(14)	13.5(14)	15.7(14)	1.1(11)	0.8(11)	1.6(11)
C26	21.0(15)	14.0(14)	16.2(14)	-2.7(11)	3.1(11)	2.2(11)
C27	15.8(14)	14.7(14)	14.3(13)	-2.5(10)	3.5(11)	-1.2(11)
C28	24.2(16)	19.5(15)	16.0(14)	-4.5(11)	6.8(12)	-1.1(12)
C29	29.9(17)	22.9(15)	13.6(13)	-1.6(12)	5.9(12)	-3.1(14)
C30	28.6(17)	16.8(15)	14.7(14)	2.7(11)	3.1(12)	-0.4(12)
C31	19.5(15)	13.7(14)	15.6(14)	0.2(11)	2.1(11)	-0.8(11)
C32	13.5(12)	11.9(12)	13.0(12)	-0.7(10)	0.9(10)	-1.2(10)

C33	24.9(16)	14.6(14)	10.6(13)	1.3(10)	0.9(11)	-1.7(12)
C34	29.1(17)	13.9(14)	17.7(15)	2.6(11)	1.6(13)	0.7(12)
C35	40(2)	16.7(16)	18.8(15)	0.1(12)	2.9(14)	6.3(14)
C36	59(3)	12.8(15)	20.9(16)	0.8(12)	7.5(16)	-2.7(16)
C37	43(2)	19.3(17)	30.0(19)	0.4(14)	13.2(16)	-10.0(15)
C38	30.8(18)	18.0(16)	23.9(16)	2.3(12)	10.8(14)	-4.6(13)
C39	16.7(13)	15.5(15)	14.2(13)	2.4(10)	3.6(10)	-1.0(11)
C40	20.5(15)	15.4(14)	17.2(14)	1.7(11)	5.7(11)	-1.4(11)
C41	28.0(18)	19.3(16)	28.2(17)	0.0(13)	13.4(14)	2.4(13)
C42	17.7(16)	29.0(19)	34.4(19)	9.2(15)	9.8(14)	5.1(13)
C43	16.6(14)	32.5(18)	28.2(17)	7.6(15)	3.3(12)	-4.6(14)
C44	19.1(15)	23.7(17)	19.5(15)	0.9(12)	2.7(12)	-4.9(12)
C45	30(2)	42(3)	57(3)	4(2)	7(2)	-2.1(18)
C46	36(2)	48(3)	54(3)	2(2)	-7(2)	-11(2)
C47	46(2)	43(3)	34(2)	2.5(18)	7.3(18)	-3(2)
C48	37(3)	92(5)	88(5)	30(4)	2(3)	-11(3)
Cl1	19.9(4)	22.0(4)	37.1(5)	2.7(3)	-11.2(3)	-1.6(3)
Cl2	34.3(4)	15.2(3)	12.8(3)	3.4(3)	0.9(3)	2.0(3)
N1	91(4)	87(4)	65(4)	1(3)	18(3)	-13(3)
N2	71(3)	69(3)	54(3)	-1(2)	13(2)	-29(3)
P1	12.7(3)	11.3(3)	11.8(3)	-0.3(2)	2.0(3)	-1.5(3)
P2	15.1(3)	10.4(3)	11.4(3)	0.9(2)	2.4(3)	-0.9(3)
Pt1	15.86(5)	12.55(5)	13.81(5)	0.26(4)	0.65(3)	1.02(4)
C51	247(15)	157(11)	336(19)	-157(12)	247(16)	-116(11)
C52	78(5)	111(9)	151(11)	-89(8)	37(6)	-27(6)
N3	192(12)	111(8)	128(8)	-48(6)	24(7)	36(7)

Table S4. Bond Lengths

Atom		Length/Å	Atom		Length/Å
C1	C2	1.399(5)	C26	C27	1.408(4)
C1	C6	1.397(5)	C27	C28	1.417(4)
C1	P1	1.805(3)	C27	C32	1.423(4)
C2	C3	1.387(5)	C28	C29	1.366(5)
C3	C4	1.375(6)	C29	C30	1.409(5)
C4	C5	1.376(6)	C30	C31	1.368(4)
C5	C6	1.398(5)	C31	C32	1.421(4)
C7	C8	1.389(4)	C33	C34	1.396(5)
C7	C12	1.395(4)	C33	C38	1.391(5)
C7	P1	1.825(3)	C33	P2	1.824(3)
C8	C9	1.398(5)	C34	C35	1.386(4)
C9	C10	1.381(5)	C35	C36	1.383(6)
C10	C11	1.387(5)	C36	C37	1.376(6)
C11	C12	1.383(4)	C37	C38	1.394(5)
C13	C14	1.420(4)	C39	C40	1.397(4)
C13	C22	1.390(4)	C39	C44	1.399(4)
C13	P1	1.833(3)	C39	P2	1.804(3)
C14	C15	1.363(4)	C40	C41	1.387(5)
C15	C16	1.412(4)	C41	C42	1.379(5)
C16	C17	1.416(4)	C42	C43	1.390(5)
C16	C21	1.418(4)	C43	C44	1.385(5)
C17	C18	1.368(5)	C45	C46	1.439(7)
C18	C19	1.410(5)	C45	N1	1.135(7)
C19	C20	1.371(5)	C47	C48	1.438(7)
C20	C21	1.419(4)	C47	N2	1.122(6)
C21	C22	1.433(4)	Cl1	Pt1	2.3518(8)
C22	C23	1.497(4)	Cl2	Pt1	2.3536(7)
C23	C24	1.389(4)	P1	Pt1	2.2448(7)
C23	C32	1.438(4)	P2	Pt1	2.2423(8)
C24	C25	1.420(4)	C51	C52	1.383(17)
C24	P2	1.834(3)	C52	N3	1.083(13)
C25	C26	1.362(4)			

Table S5. Bond Angles

Atom			Angle/°	Atom			Angle/°
C2	C1	P1	120.2(3)	C29	C28	C27	121.0(3)
C6	C1	C2	119.7(3)	C28	C29	C30	119.5(3)
C6	C1	P1	120.0(3)	C31	C30	C29	121.0(3)
C3	C2	C1	120.0(4)	C30	C31	C32	121.2(3)
C4	C3	C2	120.0(4)	C27	C32	C23	119.6(3)
C3	C4	C5	120.7(3)	C31	C32	C23	122.8(3)
C4	C5	C6	120.4(4)	C31	C32	C27	117.6(3)
C1	C6	C5	119.1(4)	C34	C33	P2	118.2(2)
C8	C7	C12	119.2(3)	C38	C33	C34	118.8(3)
C8	C7	P1	122.0(2)	C38	C33	P2	122.9(3)
C12	C7	P1	118.7(2)	C35	C34	C33	120.7(3)
C7	C8	C9	119.7(3)	C36	C35	C34	119.8(3)
C10	C9	C8	120.6(3)	C37	C36	C35	120.3(3)
C9	C10	C11	119.7(3)	C36	C37	C38	120.2(4)
C12	C11	C10	120.0(3)	C33	C38	C37	120.2(3)
C11	C12	C7	120.7(3)	C40	C39	C44	119.3(3)
C14	C13	P1	118.9(2)	C40	C39	P2	119.6(2)
C22	C13	C14	119.1(3)	C44	C39	P2	121.1(2)
C22	C13	P1	121.7(2)	C41	C40	C39	120.0(3)
C15	C14	C13	121.5(3)	C42	C41	C40	120.4(3)
C14	C15	C16	120.9(3)	C41	C42	C43	120.1(3)
C15	C16	C17	121.3(3)	C44	C43	C42	120.0(3)
C15	C16	C21	118.8(3)	C43	C44	C39	120.2(3)
C17	C16	C21	119.9(3)	N1	C45	C46	178.3(6)
C18	C17	C16	120.7(3)	N2	C47	C48	178.7(6)
C17	C18	C19	119.6(3)	C1	P1	C7	106.19(14)
C20	C19	C18	121.0(3)	C1	P1	C13	105.60(14)
C19	C20	C21	120.7(3)	C1	P1	Pt1	117.09(11)
C16	C21	C20	118.1(3)	C7	P1	C13	104.63(14)
C16	C21	C22	119.7(3)	C7	P1	Pt1	110.53(10)
C20	C21	C22	122.2(3)	C13	P1	Pt1	111.90(9)
C13	C22	C21	120.0(3)	C24	P2	Pt1	111.01(10)

C13	C22	C23	121.4(3)	C33	P2	C24	104.09(13)
C21	C22	C23	118.6(3)	C33	P2	Pt1	110.80(11)
C24	C23	C22	121.2(2)	C39	P2	C24	106.24(13)
C24	C23	C32	119.5(3)	C39	P2	C33	106.85(14)
C32	C23	C22	119.3(3)	C39	P2	Pt1	116.96(10)
C23	C24	C25	119.6(3)	Cl1	Pt1	Cl2	87.45(3)
C23	C24	P2	121.6(2)	P1	Pt1	Cl1	90.31(3)
C25	C24	P2	118.5(2)	P1	Pt1	Cl2	171.33(3)
C26	C25	C24	121.3(3)	P2	Pt1	Cl1	170.90(3)
C25	C26	C27	121.1(3)	P2	Pt1	Cl2	90.62(3)
C26	C27	C28	121.4(3)	P2	Pt1	P1	92.86(3)
C26	C27	C32	118.8(3)	N3	C52	C51	171.0(16)
C28	C27	C32	119.8(3)				

Table S6. Hydrogen Atom Coordinates (Å×10^4) and Isotropic Displacement Parameters (Å^2×10^3).

Atom	*x*	*y*	*z*	U_{eq}
H2	3026.61	201.39	4351.68	26
H3	2200.89	605.81	4993.45	37
H4	1198.93	2206.12	5053.05	44
H5	1000.53	3416.72	4479.34	42
H6	1814.16	3034.97	3827.46	28
H8	1357.42	-148.61	3744.55	28
H9	409.77	-1618.31	3383.46	36
H10	983.72	-2214.11	2722.34	33
H11	2475.99	-1312.23	2405.83	27
H12	3370.97	188.05	2747.78	22
H14	4649.77	-489.37	3342.35	17
H15	6579.75	-1027.53	3505.13	19
H17	8552.85	-804.6	3923.79	26
H18	9818.4	138.66	4426.95	30
H19	9134.68	1677.91	4761.43	28
H20	7202.39	2247.78	4603.45	21
H25	4262.97	4891.11	4065.61	19

H26	3873.48	4834.27	4780.4	20
H28	3913.94	3879.52	5483.96	23
H29	4296.08	2347.6	5896.65	26
H30	5061.04	821.49	5577.9	24
H31	5425.81	829.93	4857.26	20
H34	3250.89	5090.22	3372.83	24
H35	3265.87	6910.77	3207.46	30
H36	4990.59	7707.01	3016.05	37
H37	6712.83	6708.32	3016.24	36
H38	6719.27	4886.81	3193.87	28
H40	5650.46	1778.02	2855.5	21
H41	7459.62	996.6	2747.32	29
H42	9194.94	1550.22	3157.68	32
H43	9141.6	2915.81	3674.62	31
H44	7333.77	3665.68	3804.12	25
H46A	8788.22	10480.22	2302.09	71
H46B	10187.88	10326.06	2309.7	71
H46C	9340.97	9302.47	2304.92	71
H48A	7740.86	1146.28	797.06	109
H48B	7061.89	1892.53	427.96	109
H48C	7099.8	2216.3	932.13	109
H51A	918.41	3667.65	5559.7	346
H51B	776.48	4565.51	5920.6	346
H51C	2038.07	4019.43	5890.62	346
